# Relationship between serum vitamin D levels and the atherogenic index of plasma: a study based on NHANES database 2011–2018

**DOI:** 10.3389/fnut.2024.1468284

**Published:** 2024-11-01

**Authors:** Tingting Hu, Ying Zhang, Zhu Chen, Jun Su

**Affiliations:** ^1^Department of Clinical Laboratory, Hangzhou Women’s Hospital (Hangzhou Maternity and Child Health Care Hospital), Hangzhou, Zhejiang, China; ^2^Department of Psychosomatic Diseases, Hangzhou Seventh People's Hospital, Hangzhou, Zhejiang, China; ^3^Department of Imaging Sciences, Zhejiang Greentown Cardiovascular Hospital, Hangzhou, Zhejiang, China; ^4^School of Public Health, Hangzhou Normal University, Hangzhou Women’s Hospital (Hangzhou Maternity and Child Health Care Hospital), Hangzhou, Zhejiang, China

**Keywords:** vitamin D, arteriosclerosis index of plasma, NHANES, cardiovascular disease, saturation effect

## Abstract

**Objective:**

This study aims to investigate the relationship between serum vitamin D levels and the atherogenic index of plasma (AIP) in individuals aged 20 years and above, as well as analyze potential influencing factors.

**Methods:**

A total of 9,637 participants aged 20 years and above from the National Health and Nutrition Examination Survey (NHANES) conducted between 2011 and 2018 were included in this study. The AIP was calculated using the formula log[triglycerides (TG)/high-density lipoprotein cholesterol (HDL-C)]. Due to the skewed distribution of serum vitamin D levels in the study population, a normal transformation was performed. Weighted multivariate linear regression models were used to assess the linear relationship between the transformed serum vitamin D levels and AIP. Subgroup analysis was conducted by stratifying the data based on age, gender, and race to evaluate the stability of the relationship between serum vitamin D levels and AIP in different populations. In addition, a smooth curve fitting and generalized linear models were employed to examine the nonlinear relationship between serum vitamin D levels and AIP.

**Results:**

After controlling for confounding factors, the multivariate linear regression analysis revealed a negative correlation between serum vitamin D levels and AIP [*β* = −0.0065, 95% CI: (−0.0106, −0.0024)]. This negative correlation was significant in male participants [*β* = −0.0077, 95% CI: (−0.0142, −0.0011)], Non-Hispanic Black participants [*β* = −0.0135, 95% CI: (−0.0211, −0.0059)], as well as participants aged 40–50 [*β* = −0.0124, 95% CI: (−0.0226, −0.0022)] and 60–70 [β = −0.0118, 95% CI: (−0.0214, −0.0023)]. Furthermore, a nonlinear relationship and saturation effect were observed between the transformed serum vitamin D levels and AIP, with a turning point at 8.5617 nmol/L.

**Conclusion:**

Our study revealed a significant negative correlation and saturation effect between serum vitamin D levels and AIP.

## Introduction

Cardiovascular disease (CVD) remains a leading cause of morbidity and mortality worldwide (1). Atherosclerosis, characterized by the accumulation of arterial wall plaques, is a major contributor to the development of cardiovascular disease (2). Its progression is influenced by various factors, including abnormalities in lipid metabolism, chronic inflammation, and vitamin D levels (3, 4). Vitamin D is primarily obtained through sunlight exposure and diet (5). As a fat-soluble vitamin, vitamin D not only plays a role in regulating calcium and phosphate metabolism but also exhibits anti-inflammatory, immune-regulatory, and antioxidant effects, making it a multifunctional hormone with pleiotropic effects. Previous studies have shown that vitamin D deficiency may impact the occurrence and progression of atherosclerosis (6). Increasing circulating levels of 25-hydroxyvitamin D(25[OH]D), the major circulating form of vitamin D, has been found to effectively reduce the risk of hypertension, stroke, and myocardial infarction (7, 8). Dyslipidemia refers to an abnormal lipid/lipoprotein profile characterized by elevated total cholesterol (TC), triglycerides (TG), and low-density lipoprotein cholesterol (LDL-C) levels, along with decreased high-density lipoprotein cholesterol (HDL-C) levels, which is recognized as a significant risk factor for atherosclerosis and cardiovascular disease (9).

The atherogenic index of plasma (AIP) is a novel indicator calculated as the logarithm of the ratio of triglyceride (TG) to high-density lipoprotein cholesterol (HDL-C). It reflects the particle size and esterification rate of low-density lipoprotein cholesterol (LDL-C), which are related to lipoprotein lipase activity. Therefore, AIP is considered an important marker composed of TG and HDL-C, widely used for quantifying lipid levels and considered the optimal indicator for evaluating dyslipidemia and cardiovascular disease (CVD) (10, 11). Some studies have found AIP to be a significant and independent predictor of increased CVD risk, superior to traditional lipid parameters, and a potential biomarker for assessing the severity of coronary artery disease (12, 13). However, there is limited and conflicting research on the relationship between serum vitamin D levels and AIP. Some studies have reported a negative correlation between serum vitamin D levels and AIP, indicating that lower vitamin D levels are associated with higher AIP values (14). On the other hand, a study by Wang et al. (15) found a negative correlation between serum vitamin D concentrations and AIP in males but not in females. AIP values were higher in males with vitamin D deficiency compared to those with sufficient vitamin D levels. In order to investigate the relationship between vitamin D and AIP more accurately, we conducted this study.

The objective of this study was to elucidate the relationship between serum vitamin D levels and AIP in individuals aged 20 years and older, and further explore the influencing factors of this relationship. By analyzing a large sample dataset from NHANES 2011–2018, we aim to provide more reliable evidence to support the role of vitamin D in the prevention and treatment of atherosclerosis.

## Materials and methods

### Study population

**Figure 1:**
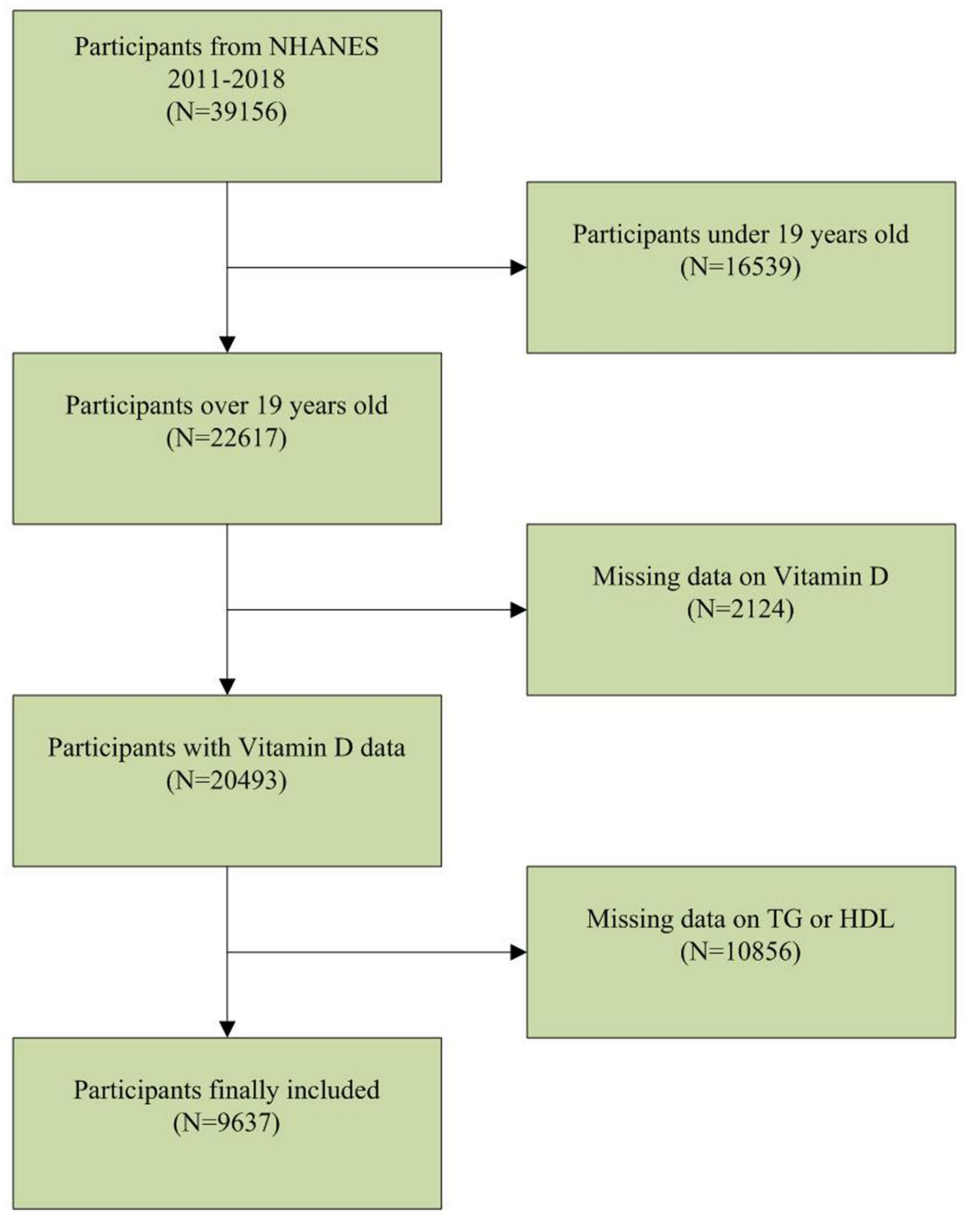
Baseline characteristics of participants.

The NHANES database is a population-based nationwide survey that provides information on population nutrition and health. The NHANES database can be publicly accessed at www.cdc.gov/nchs/nhanes. Our study utilized NHANES data from 2011 to 2018. Among the 39,156 participants, there were 16,539 individuals below the age of 20, 2,124 with missing serum vitamin D data, and 10,856 with missing TG or HDL data. After applying these exclusion criteria, a total of 9,637 participants were included in the clinical analysis (Figure 1).

### Study variables

**Figure 2:**
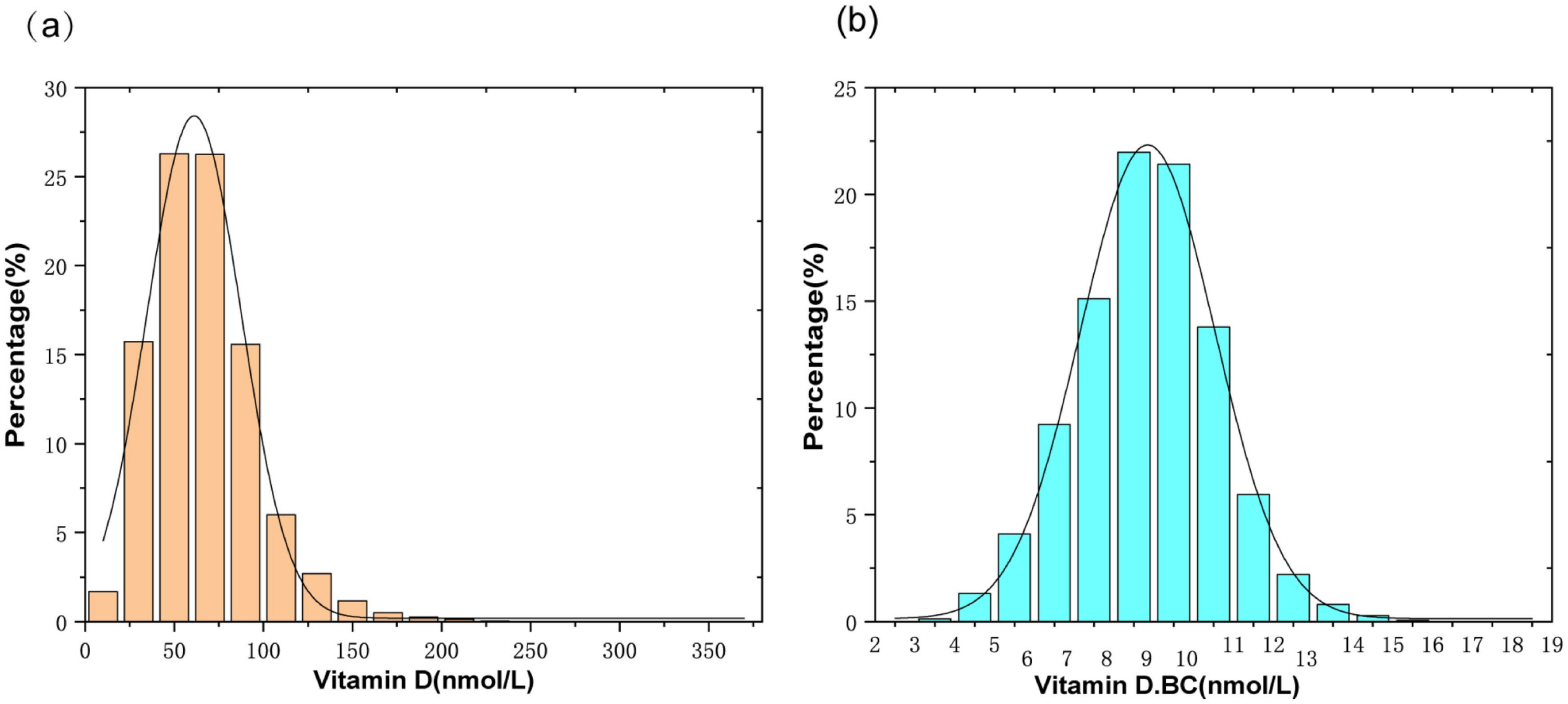
**(A)** Histogram of serum vitamin D distribution; **(B)** Histogram of serum vitamin D distribution after normalization transformation(Vitamin D.BC).

The independent variable in this study is serum Vitamin D. Due to the skewed distribution of serum Vitamin D in the study population (Figure 2A), a normal transformation was applied to serum Vitamin D in the article (Figure 2B). The dependent variable is the calculated plasma atherogenic index (AIP), which is computed using the formula AIP = log (TG [mg/dL]/HDL-C [mg/dL]). The following variables were included as covariates in the clinical analysis: age, gender, race, ratio of family income to poverty (PIR), body mass index (BMI), alanine aminotransferase (ALT), aspartate aminotransferase (AST), blood urea nitrogen (BUN), serum creatinine (Scr), TC, alkaline phosphatase (ALP), Calcium (Ca), and Phosphorus (P). The examination section related to clinical and laboratory evaluations was provided by well-trained medical experts. Detailed procedures and measurement methods for each variable can be found at www.cdc.gov/nchs/nhanes.

### Statistical analysis

All analyses were conducted using the weights from the NHANES examination sample, and the baseline characteristics of all participants included in the final analysis were described as mean ± standard deviation (continuous variables) or percentage (categorical variables). A weighted multivariable linear regression model was used to assess the linear relationship between serum Vitamin D after normal transformation and AIP, and subgroup analyses were performed to evaluate the linear relationship between serum Vitamin D and AIP in different populations by stratifying for age, gender, and race. Additionally, a smoothed curve fitting and generalized linear models were employed to investigate the non-linear relationship between serum Vitamin D and AIP. The inflection point (if it existed) was calculated using a two-segment linear regression model with a recursive algorithm. A *p* value <0.05 was considered statistically significant. We utilized EmpowerStats (http://www.empowerstats.com, X&Y Solutions, Inc., Boston, MA) and the statistical software package R (http://www.Rproject.org, The R Foundation) for the modeling process. Frequency distribution plots for serum Vitamin D and serum Vitamin D after normal transformation were generated using Origin (version: 2024).

## Results

### Baseline characteristics of participants

After applying the inclusion and exclusion criteria, a total of 9,637 participants met the criteria. The population characteristics, weighted according to the quartiles of serum Vitamin D after normal transformation (Q1: 6.65 ± 0.82 nmol/L; Q2: 8.31 ± 0.34 nmol/L; Q3: 9.40 ± 0.32 nmol/L; Q4: 10.99 ± 0.93 nmol/L), are presented in Table 1. Significant differences were observed in age, gender, race, PIR, BMI, ALT, BUN, Scr, TC, TG, HDL, LDL, ALP, and Ca among different groups based on serum Vitamin D quartiles (Q1–Q4). Compared to the lowest quartile, individuals in the highest quartile were more likely to be older, female, have a higher PIR, a higher proportion of Non-Hispanic Whites, and higher levels of BUN, Scr, TC, HDL, LDL, and Ca. Conversely, they exhibited lower BMI, ALT, TG, and ALP levels (Table 1).

**Table 1:** Weighted characteristics of 9,637 participants included in this study.

Characteristics	Vitamin D(nmol/L)				
	Q1	Q2	Q3	Q4	*p* value
	*N* = 2,405	*N* = 2,410	*N* = 2,399	*N* = 2,423	
Age	42.66 ± 15.78	43.93 ± 16.24	47.68 ± 16.47	55.21 ± 16.22	<0.0001
Sex (%)					<0.0001
Male	48.06	56.12	53.86	39.55	
Female	51.94	43.88	46.14	60.45	
Race (%)					<0.0001
Mexican American	16.71	13.14	6.64	2.34	
Other Hispanic	6.13	9.33	7.87	3.19	
Non-Hispanic White	37.98	56.80	71.16	83.81	
Non-Hispanic Black	28.00	10.00	6.05	4.20	
Other race	11.19	10.73	8.28	6.46	
PIR	2.35 ± 1.57	2.72 ± 1.63	3.08 ± 1.64	3.35 ± 1.60	<0.0001
BMI (kg/m^2^)	31.23 ± 8.37	29.86 ± 7.08	29.02 ± 6.56	27.90 ± 6.19	<0.0001
ALT (U/L)	25.47 ± 18.66	26.40 ± 19.71	24.15 ± 15.89	23.39 ± 14.72	<0.0001
AST (U/L)	25.04 ± 17.08	24.63 ± 22.40	24.08 ± 13.49	24.88 ± 13.54	0.2111
BUN (mg/dL)	4.40 ± 2.07	4.75 ± 1.75	5.05 ± 1.72	5.36 ± 2.09	<0.0001
Scr (mg/dL)	0.85 ± 0.57	0.86 ± 0.37	0.88 ± 0.29	0.91 ± 0.39	<0.0001
TC (mg/dL)	186.76 ± 41.20	186.85 ± 41.00	191.84 ± 40.34	194.76 ± 41.47	<0.0001
TG (mg/dL)	121.76 ± 136.28	125.27 ± 110.17	120.50 ± 84.83	115.10 ± 84.36	0.0035
HDL (mg/dL)	51.68 ± 15.08	50.53 ± 14.18	53.47 ± 15.39	59.78 ± 18.71	<0.0001
LDL (mg/dL)	111.48 ± 34.85	111.72 ± 35.56	114.51 ± 34.37	112.47 ± 36.03	0.0143
ALP (U/L)	72.80 ± 25.83	69.72 ± 29.69	67.64 ± 22.84	66.58 ± 21.53	<0.0001
Ca (mg/dL)	9.28 ± 0.34	9.29 ± 0.33	9.34 ± 0.33	9.37 ± 0.37	<0.0001
P (mg/dL)	3.65 ± 0.60	3.63 ± 0.53	3.64 ± 0.54	3.66 ± 0.54	0.2298
Vitamin D (nmol/L)	34.50 ± 8.04	54.75 ± 4.95	72.12 ± 5.56	105.26 ± 23.43	<0.0001
Vitamin D.BC	6.65 ± 0.82	8.31 ± 0.34	9.40 ± 0.32	10.99 ± 0.93	<0.0001
AIP	0.29 ± 0.35	0.32 ± 0.34	0.29 ± 0.34	0.23 ± 0.33	<0.0001

Mean ± SD for continuous variables: the *p* value was calculated by the weighted linear regression model. (%), for categorical variables: the *p* value was calculated by the weighted chi-square test.

Q, Quartile; PIR, Ratio of family income to poverty; BMI, Body mass index; ALT, Alanine aminotransferase; AST, Aspartate aminotransferase; BUN, Blood urea nitrogen; SCr, Serum creatinine; TC, Total cholesterol; TG, Triglyceride; HDL-C, High-density lipoprotein cholesterol; LDL-C, Low-density lipoprotein cholesterol; ALP, Alkaline phosphatase; ALP, Alkaline phosphatase; Vitamin D, 25OHD2 + 25OHD3; Vitamin D.BC, Vitamin D after normalization transformation; AIP, Atherogenic index of plasma.

### Association between serum vitamin D and AIP

Table 2 presents the relationship between serum Vitamin D and Atherogenic Index of Plasma (AIP). Three weighted multivariate linear regression models were constructed. In the unadjusted model, there was a negative correlation between serum Vitamin D and AIP [*β* = −0.0169, 95% CI: (−0.0209, −0.0129)]. After controlling for confounding factors, this negative correlation persisted in Model 2 [β = −0.0258, 95% CI: (−0.0301, −0.0215)] and Model 3 [β = −0.0065, 95% CI: (−0.0106, −0.0024)]. When serum Vitamin D was converted from a continuous variable to a categorical variable (quartiles), individuals in the highest quartile had an AIP that was 0.0266 lower than those in the lowest quartile.

**Table 2:** Association between VitaminD.BC (nmol/L) and AIP.

Exposure	Model 1, [β (95% CI)]	Model 2, [β (95% CI)]	Model 3, [*β* (95% CI)]
VitaminD.BC (continuous)	−0.0169 (−0.0209,−0.0129)	−0.0258 (−0.0301,−0.0215)	−0.0065 (−0.0106,−0.0024)
Vitamin D.BC (quartile)			
Quartile 1	Reference	Reference	Reference
Quartile 2	0.0274 (0.0064, 0.0485)	−0.0153 (−0.0360, 0.0054)	0.0063 (−0.0127, 0.0252)
Quartile 3	−0.0028 (−0.0232, 0.0176)	−0.0536 (−0.0742, −0.0330)	0.0043 (−0.0147, 0.0234)
Quartile 4	−0.0627 (−0.0824, −0.0429)	−0.1098 (−0.1307, −0.0889)	−0.0266 (−0.0462, −0.0070)
*p* for trend	<0.001	<0.001	0.002

Model 1: no covariates were adjusted. Model 2: age and gender were adjusted. Model 3: age, gender, race, PIR, BMI, ALT, AST, BUN, Scr, TC, LDL, ALP, Ca, and P were adjusted.

### Subgroup analysis

Subgroup analyses were performed in this study to assess the stability of the relationship between Atherogenic Index of Plasma (AIP) and serum Vitamin D across different population backgrounds. The results showed a significant negative correlation between serum Vitamin D and AIP among male participants [−0.0077 (−0.0142, −0.0011)]. When stratified by race, Non-Hispanic Black participants exhibited a significant negative correlation between serum Vitamin D and AIP [−0.0135 (−0.0211, −0.0059)]. Among different age groups, participants aged 40–50 years [−0.0124 (−0.0226, −0.0022)] and 60–70 years [−0.0118 (−0.0214, −0.0023)] demonstrated a significant negative correlation between serum Vitamin D and AIP. Other factors did not significantly influence the relationship between serum Vitamin D and AIP (Table 3).

**Table 3:** Association between Vitamin D.BC and AIP stratified by sex, race and age.

	Model 1, β (95% CI) *p* value	Model 2, β (95% CI) *p* value	Model 3, β (95% CI) *p* value
Stratified by gender			
Male	−0.0149 (−0.0214, −0.0084)***	−0.0275 (−0.0347, −0.0203)***	−0.0077 (−0.0142, −0.0011)*
Female	−0.0112 (−0.0159, −0.0064)***	−0.0249 (−0.0302, −0.0196)***	−0.0024 (−0.0075, 0.0028)
Stratified by race			
Mexican American	−0.0019 (−0.0146, 0.0107)	−0.0101 (−0.0229, 0.0026)	0.0020 (−0.0095, 0.0136)
Other Hispanic	−0.0094 (−0.0250, 0.0062)	−0.0120 (−0.0273, 0.0033)	0.0086 (−0.0057, 0.0229)
Non-Hispanic White	−0.0330 (−0.0400, −0.0259)***	−0.0311 (−0.0383, −0.0240)***	−0.0063 (−0.0130, 0.0004)
Non-Hispanic Black	−0.0183 (−0.0256, −0.0110)***	−0.0249 (−0.0325, −0.0174)***	−0.0135 (−0.0211, −0.0059)***
Other Race	−0.0082 (−0.0180, 0.0015)	−0.0138 (−0.0241, −0.0036)**	−0.0050 (−0.0149, 0.0049)
Stratified by age			
Aged<30	−0.0142 (−0.0249, −0.0035)**	−0.0220 (−0.0336, −0.0104)***	−0.0066 (−0.0170, 0.0038)
30 ≤ aged<40	−0.0149 (−0.0258, −0.0041)**	−0.0190 (−0.0306, −0.0074)**	−0.0047 (−0.0152, 0.0058)
40 ≤ aged<50	−0.0246 (−0.0354, −0.0138)***	−0.0313 (−0.0426, −0.0201)***	−0.0124 (−0.0226, −0.0022)**
50 ≤ aged<60	−0.0281 (−0.0381, −0.0181)***	−0.0302 (−0.0404, −0.0199)***	−0.0069 (−0.0171, 0.0033)
60 ≤ aged<70	−0.0312 (−0.0401, −0.0224)***	−0.0311 (−0.0401, −0.0221)***	−0.0118 (−0.0214, −0.0023)*
70 ≤ aged<80	−0.0142 (−0.0233, −0.0051)**	−0.0148 (−0.0239, −0.0057)*	−0.0016 (−0.0105, 0.0072)

In subgroup analyses stratified by gender, race, age, Model 1: no covariates were adjusted. Model 2: age and sex were adjusted. Model 3: age, gender, race, PIR, BMI, ALT, AST, BUN, Scr, TC, LDL, ALP, Ca, and P were adjusted.*p < 0.05, **p < 0.01, ***p < 0.001. *p* < 0.05 was considered statistically significant.

### Non-linearity and saturation effect analysis between serum vitamin D and AIP

**Figure 3:**
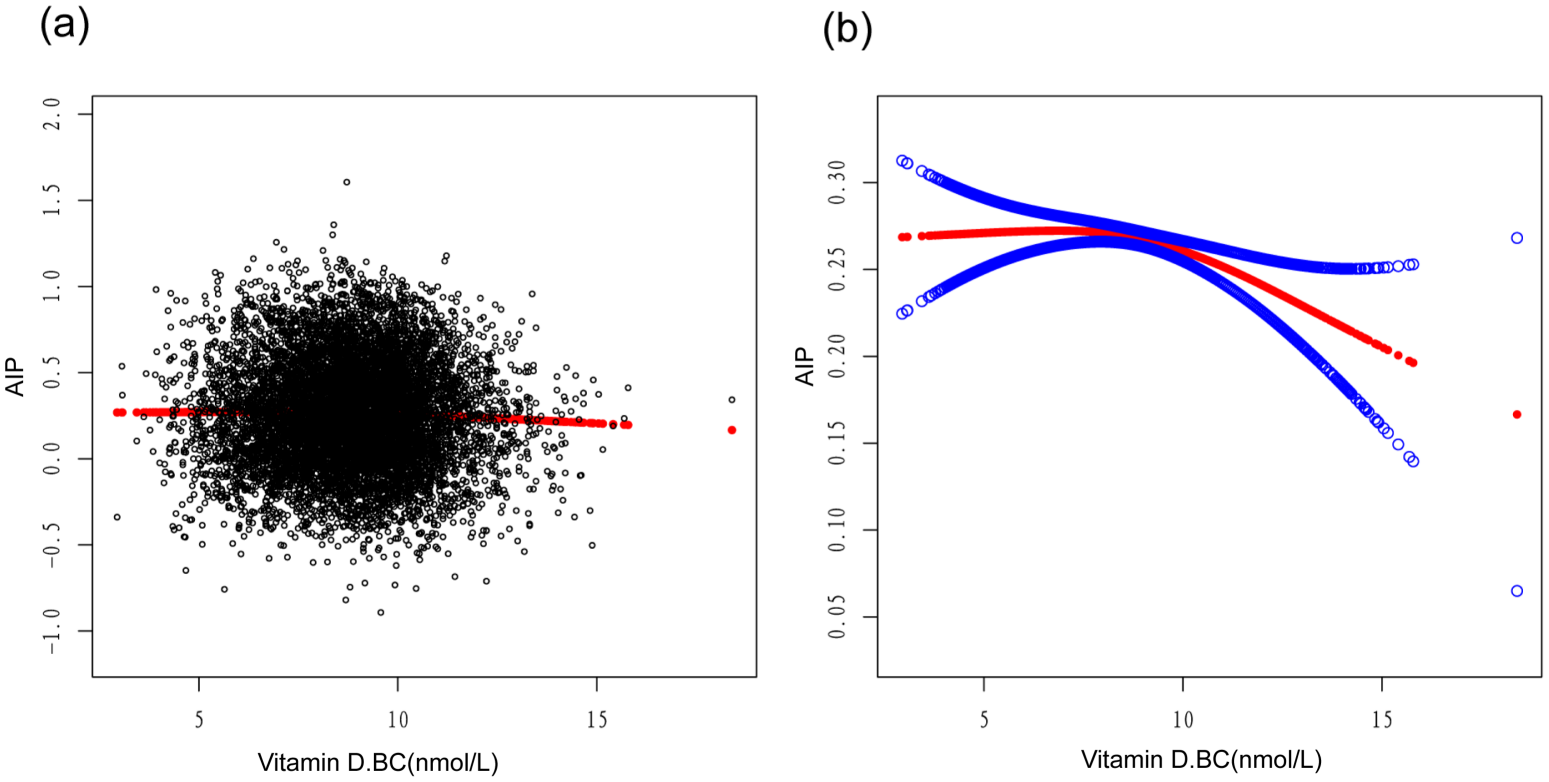
Association between vitamin D.BC and AIP (The solid red line represents the smooth curve fit between variables. Blue bands represent the 95% confidence interval from the fit).

A smoothed curve fitting was used to describe the non-linear association and saturation phenomenon between serum Vitamin D and Atherogenic Index of Plasma (AIP) (Figure 3). The results showed that the saturation point for the relationship between serum Vitamin D (after undergoing a normal transformation) and AIP in all participants was 8.5617 nmol/L. When the transformed serum Vitamin D was below 8.5617 nmol/L, the effect size was 0.0130; whereas when the transformed serum Vitamin D exceeded 8.5617 nmol/L, the effect size changed to −0.0184 (Table 4).

**Table 4:** Saturation effect analysis of SerumVitaminD.BC (nmol/L) on AIP.

AIP	Model: saturation effect analysis [β (95% CI) *p* value]
SerumVitaminD.BC turning point (K)	8.5617
<K, effect1	0.0130 (0.0044, 0.0217) 0.0032
>K, effect2	−0.0184 (−0.0245, −0.0122) <0.0001
Log-likelihood ratio	<0.001

Age, gender, race, PIR, BMI, ALT, AST, BUN, Scr, TC, LDL, ALP, Ca, and P were adjusted.

## Discussion

In our study, we found a negative correlation between serum vitamin D levels and AIP. Further subgroup analysis revealed significant associations between serum vitamin D levels and AIP in male participants, non-Hispanic black individuals, and those aged between 40–50 and 60–70 years. Interestingly, we observed an inverted L-shaped relationship between logarithmically transformed serum vitamin D levels and AIP, with a turning point at 8.5617 nmol/L.

Cardiovascular disease (CVD) poses a significant threat to human health, with high global incidence, mortality, and disability rates (16). AIP has been identified as one of the strongest biomarkers for predicting CVD risk (17). AIP reflects the balance between pro-atherogenic lipids such as triglycerides and anti-atherogenic lipids like high-density lipoprotein cholesterol (18). It can serve as an adjunct to individual lipid profiles. AIP is a better determinant of HDL-C particle fractionation than conventional lipid parameters (19, 20). Studies have shown that higher AIP values are associated with an increased risk of coronary artery disease (CAD) (21). In recent years, an increasing number of studies have demonstrated an association between low 25(OH)D levels and increased cardiovascular disease risk and all-cause mortality (22, 23). Ge et al. (24) found in their study among rural Chinese population that serum 25(OH)D3 concentration was correlated with lipid levels, with varying associations between individuals with normal lipid levels and those with abnormal lipid levels; as serum 25(OH)D3 levels increased, the incidence of lipid abnormalities decreased. A cross-sectional study conducted among middle-aged and elderly Chinese population found a positive correlation between vitamin D deficiency and abnormal lipid profiles and AIP (25). Our study also revealed similar findings in individuals aged 20 and above.

In this study, we found a significant correlation between serum vitamin D levels and AIP. After adjusting for confounding factors, we observed a significant negative correlation between serum vitamin D levels and AIP in males. However, in females, our results showed a *p* value greater than 0.05, indicating no significant relationship between serum vitamin D levels and AIP after adjusting for confounding factors. This finding is consistent with some previous studies. Wang et al. (15) reported a negative correlation between serum 25(OH)D concentration and AIP in males but not in females. Furthermore, males with vitamin D deficiency had higher AIP values compared to males with sufficient vitamin D. Naganuma et al. (26) reported that low serum 25(OH)D levels were associated with increased atherosclerosis risk in adolescent boys but not in girls. Several factors may contribute to these gender-specific differences. First, it could be related to hormonal changes. Hormones have profound effects on lipid metabolism (27), which could have different impacts on the relationship between vitamin D and AIP in males and females. Sex hormones play an important role in the regulation of lipid metabolism. A sex-stratified meta-analysis identified lipid-related loci showing sex-biased effects on both autosomes and the X chromosome, with associations with the pleiotropy of sex hormones, highlighting the important role of sex hormone regulation in lipid metabolism (28). These hormones may interact with the vitamin D signaling pathway, leading to gender-specific effects on AIP. Secondly, vitamin D signaling has multiple effects outside the skeletal system, including regulation of cell proliferation, immune and muscle function, skin differentiation and reproduction, as well as vascular and metabolic properties (29). These effects may manifest differently in males and females, resulting in differential associations between serum vitamin D levels and AIP in both sexes. Lastly, there are differences in dietary patterns, exercise habits, sunlight exposure, and other factors between males and females, which may influence the synthesis and absorption of vitamin D. Therefore, these behavioral and lifestyle differences between male and female populations may modulate the relationship between serum vitamin D levels and AIP.

We conducted a stratified analysis based on race and found a significant negative correlation between serum vitamin D and AIP among non-Hispanic Black individuals, while no such phenomenon was observed in other races. This may be related to genetic variations that affect vitamin D metabolism. In a cross-sectional study of multi-ethnic populations with atherosclerosis (MESA), significant racial differences were found in vitamin D metabolism indicators. Compared to Black participants, White participants had significantly higher concentrations of 25-hydroxyvitamin D in their serum. The ratios of circulating vitamin D metabolites indicated lower *CYP27B1* activity and higher *CYP24A1* activity among White participants. Differences in vitamin D-binding globulin haplotypes were also observed (30). These genetic variations may lead to different ways of metabolizing and utilizing vitamin D among non-Hispanic Black participants compared to other races. Therefore, the relationship between vitamin D and AIP may exhibit different patterns.

When stratified by age, we found a significant negative correlation between serum vitamin D levels and plasma atherogenic index of plasma (AIP) among participants aged 40–50 and 60–70. With increasing age, there are various changes in the metabolism and activity of vitamin D. The ability of the skin to produce vitamin D3 decreases with age, reducing by 13% every decade (31). The resistance of the intestines to 1,25-dihydroxyvitamin D increases, affecting calcium absorption in the gut. Among various organs involved in calcium metabolism, the number of vitamin D receptors decreases with age, and the activity of 1α-hydroxylase decreases mainly due to declining kidney function, leading to reduced activation of vitamin D (32). Vitamin D deficiency is common in the elderly population as a result. Age-related factors also include changes in hormone and bone morphogenetic protein levels. In conclusion, this significant negative correlation may be attributed to age-related changes in vitamin D metabolism, cumulative effects of vitamin D deficiency, alterations in lipid metabolism, and complex interactions with other age-related factors (33). Further research is needed to understand the exact mechanisms and clinical significance of this age-specific relationship.

In our study, we employed a smooth curve fitting to describe the non-linear association and saturation phenomenon between serum vitamin D and AIP. The saturation effect value of 8.5617 nmol/L may have physiological significance in the relationship between vitamin D and AIP. When the serum vitamin D level, transformed into a normal distribution, is below this threshold, its regulatory effect on AIP is limited, while beyond this threshold, vitamin D may exert a stronger negative regulatory effect. The interpretation of these findings may also need to consider the metabolism and mechanisms of action of vitamin D. Vitamin D mediates its biological effects in cells by binding to vitamin D receptors. The saturation phenomenon may reflect the saturation or regulatory mechanism of these receptors, resulting in a non-linear relationship and the manifestation of a saturation effect for vitamin D. Similar studies have found a U-shaped association between serum 25(OH)D levels and CVD risk, suggesting a non-linear relationship between vitamin D and CVD prevalence (34). It should be noted that although we observed the saturation effect between serum vitamin D and AIP, further research is still needed to determine the optimal level of vitamin D. An animal model experiment showed that high-dose vitamin D, as an adjunct to simvastatin therapy, was superior to omega-3 levels in improving TG, HDL, and AIP (35). Additionally, maintaining an appropriate serum level of vitamin D appears to be crucial for calcium homeostasis and cardiovascular risk, blood pressure regulation, stroke incidence, metabolic syndrome, and peripheral arterial disease. Vitamin D exerts beneficial effects on the cardiovascular system by reducing the activity of the renin-angiotensin-aldosterone system (RAAS), lowering blood pressure, and possessing anti-inflammatory, anti-proliferative, anti-hypertensive, anti-fibrotic, anti-diabetic, and anti-thrombotic properties (36). These potential benefits further underscore the significance of determining the optimal level of vitamin D and suggest that vitamin D may play a vital role in the prevention and treatment of cardiovascular diseases.

The investigation of the non-linear association between vitamin D and AIP may contribute to a better understanding of the biological effects of vitamin D and its impact on cardiovascular health. One of the major strengths of this study was the utilization of the NHANES database, which provided a large representative sample of the general population. By employing rigorous statistical analysis and adjusting for confounding factors, we were able to establish a strong association between serum vitamin D levels and AIP. However, there were several limitations to our study. Firstly, the cross-sectional design of NHANES limited our ability to establish causality. Secondly, reliance on self-reported data may have introduced recall bias. Thirdly, our findings may not be generalizable to populations beyond the NHANES sample. Future prospective studies and clinical trials are necessary to confirm our findings and explore underlying mechanisms.

## Conclusion

In conclusion, our study uncovered a negative correlation between serum vitamin D levels and AIP, suggesting a potential protective role against atherosclerosis and cardiovascular diseases. Subgroup analyses stratified by gender, race, and age revealed interesting variations in the associations. These findings highlight the significance of optimizing vitamin D status as a prospective preventive strategy for cardiovascular diseases, including atherosclerosis. Further research, including prospective studies and clinical trials, is warranted to validate our findings and elucidate the underlying mechanisms.

## Data Availability

Publicly available datasets were analyzed in this study. This data can be found here: www.cdc.gov/nchs/nhanes.
